# Effects of visual aid on state anxiety, fear and stress level in patients undergoing endoscopy: a randomized controlled trial

**DOI:** 10.1080/07853890.2023.2191000

**Published:** 2023-04-20

**Authors:** Ali Asad Khan, Anam Ali, Ali Salman Khan, Yasir Shafi, Mohsin Masud, Fatima Irfan, Sajid Abaidullah

**Affiliations:** a North Medical Ward, King Edward Medical University, Lahore, Pakistan; b Department of Developmental and Behavioral Pediatrics, University of Child Health Sciences, The Children’s Hospital (UCHS-CH), Lahore, Pakistan; c Acute Medical Unit, North Manchester General Hospital, Manchester, England

**Keywords:** Endoscopy, randomized controlled trial, counselling, visual aid, anxiety

## Abstract

**Objectives:**

The present study aimed to calculate the estimated size and confidence interval for the effects of adding visual aid to counselling on anxiety, stress and fear of patients undergoing upper gastrointestinal endoscopy. The secondary aim was to calculate confidence interval for endoscopy-related variables that predict which patients are likely to benefit from visual aids.

**Method:**

In a randomized, single-blind, two arm, parallel group, superiority trial, 232 consecutive patients who were scheduled to undergo either gastroscopy or colonoscopy were randomly divided into two intervention groups; counselling with video of endoscopic procedure and counselling with no-video (*n* = 116 in each group). Primary outcome was anxiety and secondary outcomes were stress and fear.

**Results:**

One-way ANCOVA showed that there was significant between group differences of anxiety, stress and fear after controlling for the effect of covariates. Planned contrasts revealed that counselling along with visual aid of endoscopy procedure significantly decreased anxiety [Mean difference at post; −4.26 (−4.47, −4.05), *p* < .001, partial *η*^2^ = 0.88], stress [−5.35 (−5.63, −5.07), *p* < .001, partial *η*^2^ = 0.86] and fear [−2.82 (−2.97, −2.67), *p* < .001, partial *η*^2^ = 0.86] compared to counselling alone. Linear regression showed that gender, nature of complaints and concern over seniority of endoscopist were significant negative predictors, however, satisfaction on briefing of endoscopy procedure was significant positive predictor of outcome variables in visual aid condition.

**Conclusion:**

The increase in anxiety, acute stress and fear related to endoscopic procedures can be alleviated with psychological counselling coupled with visual aids before the procedure. Visual aid could lead to supplementary benefits in reducing anxiety scores.

**Trial Registration:**

ClinicalTrial.gov Number: NCT05241158. Registered 16/11/2022; https://clinicaltrials.gov/ct2/show/NCT05241158

**KEY MESSAGES**
- Counselling along with visual aid of endoscopy procedure significantly decreased anxiety, stress and fear as compared to counselling alone.
- Male patients were less stressed after visual aid intervention as compared to female patients. Patients who had chronic GI symptoms were less stressed after visual aid intervention as compared to those who had acute GI symptoms. Patients who had concern over seniority of endoscopist were less stressed after visual aid intervention as compared to those who had no concerns over seniority.
- Satisfaction on briefing of endoscopy procedure was significant positive predictor of stress and fear.

## Introduction

Anxiety is an organic reaction manifested by physical (palpitations, shortness of breath, tremors) and emotional (apprehension, irritability, restlessness) symptoms [1]. Anxiety is classified as a psychophysiological state (state anxiety) or a personality trait (trait anxiety) [2]. Medical conditions can trigger state anxiety episodes caused by the symptoms of a disease, adverse effects of medicines, any diagnostic or interventional procedures (medical, surgical or dental) and stress from a serious or chronic medical illness [3].

Endoscopic procedures (gastroscopy, colonoscopy, Endoscopic retrograde cholangiopancreatography, Endoscopic ultrasound) are associated with an upstroke in anxiety levels due to the fear of diagnosis of a lifelong or potentially fatal medical condition [4]. Ersöz et al. studied the anxiety levels in patients undergoing endoscopic procedures (gastroscopy and colonoscopy) and found a significant increase in state anxiety prior to gastroscopy and colonoscopy [5]. Other studies also reported similar findings that investigated the impact of surgical interventions on state anxiety or acute stress levels in patients regardless of the level of severity or type of endoscopic procedure conducted on the patients [6,7]. Therefore, studies investigated the role of psychological preparation of the patients before endoscopic procedures in which patients were assigned to control and experimental groups (behavior intervention). A significant reduction in anxiety scores was found due to psychological preparation in the experimental group [4,8].

Özkan and Fındık conducted a randomized controlled trial (RCT) to see the effect of providing information about clonoscopy on patient’s anxiety level related to procedure and divided into experimental (written and oral information was provided) and control group. They reported that anxiety of patients in intervention group was reduced as compared to control group [9]. Lu et al. also conducted RCT to see the effect of receiving broucher information or conjuctive interactive information of gastroscopy on anxiety level of patients and found that information through mobile social media application reduce pre-gastroscopic anxiety and discomfort in patients [10].

Murugesan et al. conducted RCT to see the impact of video instruction on patient’s anxiety levels who underwent colonoscopy. Patients were divided into two groups; video or control (verbal) group. They concluded that patients who were shown information video before colonoscopy had low anxiety levels [11]. Similarly, other randomized controlled trials were done to see the efficacy of music on patient’s anxiety who underwent endoscopy. It was found that there was significant difference in post-anxiety levels of patients who listened to music as compared to control group (who didn’t listen to music) [12,13].

Sogabe et al. conducted RCT to study the effects of audio-visual distraction in patients who underwent upper GI endoscopy. Patients were divided into four groups; control group, auditive group (listened to healing music), visual group (watched silent natural image) and combined audio-visual (watched natural image while listening to music) group. Results revealed that pulse rate at post-distraction and post-esophagogastroduodenoscopy were significantly lower in three intervention groups as compared to control group [14].

A number of RCTs have been conducted who studied the efficacy of visual distraction [15], audio distraction (music therapy) [12,13], or combination of both in patients of gastrointestinal diseases [14,16]. As per researcher’s knowledge, there has been no randomized controlled trial that studied the impact of showing an animated video of endoscopy before the procedure on anxiety of patients undergoing upper GI endoscopy. Therefore, the present study aimed to study the effects on anxiety of adding visual aids to counselling in patients undergoing gastrointestinal endoscopy. The secondary aim was to calculate treatment effect sizes for patient-reported outcome measures such as stress and fear for upper GI endoscopy. Moreover, it was also aimed to calculate confidence interval for endoscopy-related variables that predict which patients are likely to benefit from visual aids.

### Hypotheses

- There was no significant difference in average post-intervention anxiety scores between visual aid and no visual aid condition after controlling for covariates (pre-intervention anxiety scores).
- There was no significant difference in average post-intervention stress and fear scores between visual aid and no visual aid condition after controlling for covariates.
- Endoscopy-related variables are likely to have an impact on counselling with visual aid condition.

## Methods

### Study design

This study was designed as randomized, assessor-blind, parallel group, superiority trial. It was conducted at Mayo Hospital, Lahore. Ethical committee of King Edward Medical University approved the study protocol (Ref No. 179/RC/KEMU) and it was registered in ClinicalTrial.gov, number: NCT05241158.

### Sample and randomization process

A total of 274 consecutive patients, scheduled to undergo either a gastroscopy or a colonoscopy (diagnostic or therapeutic) at the outpatient department of Medical Unit of Mayo Hospital from October 2018 to February 2020, participated in the study. Written informed consent was taken from every patient prior to participation in the study. They were debriefed about the nature and purpose of the study and their role. Forty-two patients were excluded from the study because they met any of the following criteria: (1) diagnosed cases of psychiatric illnesses; (2) history of previous endoscopy; (3) undergoing emergency endoscopy (whether diagnostic or therapeutic); (4) end stage renal disease; (5) hearing or visual difficulty; (6) senile dementia; (7) pregnant or diagnosed cases of malignancy; (8) signs of hepatic encephalopathy.

**Figure 1.**
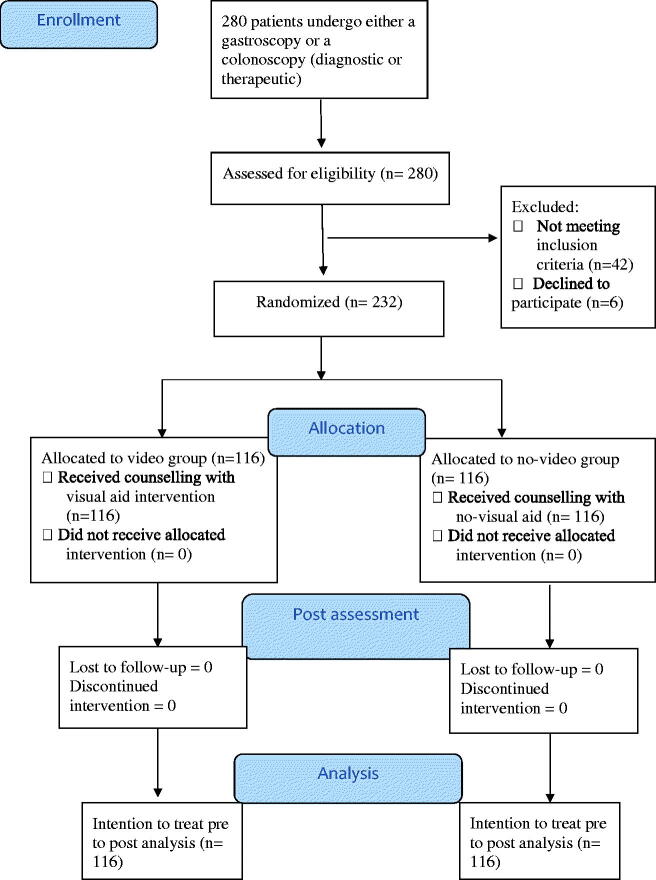
CONSORT flow chart of the participants through the trial.

Two hundred thirty-two eligible patients who were enrolled in the study (see Figure 1), received regular instructions regarding gut-preparation at the time of endoscopy scheduling and were also provided with written clear instructions. A gastroenterologist provided information about endoscopy, including the exact preparation instructions and information on the importance of bowel preparation and the adverse effects of the agents used.

For gastroscopy, patients were instructed to stop eating 6 h before the procedure and stop drinking all kinds of liquids 2 h prior to the procedure. For colonoscopy, patients were given bowel preparation kits that contained Polyethylene Glycol (PEG 3350) and electrolytes. The kits were to be mixed in 1 liter of water and consumed 24 h before the procedure. Patients were instructed to stop eating once they used the kit and use clear liquids (water or oral rehydrating solutions) till the time they reported for the procedure.

Patients were admitted to Medical Unit a day before endoscopy. They were shifted to endoscopy floor in the morning after a longer than 12 h fasting period. Demographic questionnaire, Depression, Anxiety and Stress Scale-42 (DASS-42) and Visual analogue scale (VAS) were administered by psychologist as baseline in a separate room. 232 patients were then randomly allocated between two groups (group 1 watched animated video of endoscopy procedure, however, group 2 didn’t watch any video) on a 1:1 ratio using permuted block randomization with a fixed block sizes of four, by a house officer who wasn’t involved in providing intervention to any group or in assisting any of the endoscopy procedures and was responsible to break the code in case of adverse event. The allocation sequence to the study conditions was kept concealed from psychologists measuring outcome variables, using sequentially numbered, opaque and sealed envelopes. The nature of the investigation required patients to be aware of what group they were in, so, the patients were not blinded to treatment group allocation. Patients were told not to inform anyone about their allocation. Patients were assured about confidentiality of their responses and their identities. They were also told that information obtained from them will only be used for academic and research purposes. No financial and other inducements were offered to the participants that were likely to coerce participation.

### Intervention group; counselling with visual aid

First of all, counselling was done in which patients were given a briefing about the procedure; the use of local anesthetic along with potential benefits and side effects of the drug. The steps of the endoscopy procedure (gastroscopy or colonoscopy) were explained in detail including the position, intubation, biopsy or intervention wherever applicable, extubation and post-procedure observation period in the recovery room. Detailed instructions were provided regarding post-procedural care, introduction of diet & follow-up. In addition to that, video education was provided. During video education, patients watched a 2-min video of the respective procedure. Video showed the animation of a gastroscopy or colonoscopy procedure with a voice over explaining all the steps of the procedure in addition to the pre- and post-procedure precautions to be observed [17].

### Intervention group; counselling with no-visual aid

Patients in the no-visual aid group, counselling was done exactly in the same way as of visual aid group. However, no video was shown to the patients of this group.

Intervention was provided to all participants in a private room by a gastroenterologist with experience of 10 years. Post-intervention assessment using DASS-42 and VAS was done immediately after intervention, but prior to endoscopy procedure, by psychologist who was blinded to group allocation. Patients then prepared for the procedure and underwent endoscopy. Endoscopies were carried out by trained doctors (endoscopists) with a minimum experience of 1000 endoscopic procedures. All methods and procedures of gastroscopy and colonoscopy were performed in accordance with the relevant guidelines issued by American College of Gastroenterology. Findings were noted in respective areas after endoscopy. Post-endoscopy, eight patients were shifted to intensive care due to unstable hemodynamic condition (variceal bleeding from esophageal or gastric varices, bleeding gastric or duodenal ulcers) found during endoscopy. Patients were retained in the recovery room for 2 h after the procedure and vital signs were monitored along with any evidence of bleeding through the mouth, altered level of consciousness or signs of shock. Patients were discharged in stable condition.

### Outcomes

The primary outcome measure was anxiety (as scored using Depression, Anxiety and Stress Scale-42; DASS-42). The secondary outcome measures were stress and fear, as scored using DASS-42 and Visual analogue scale (VAS) respectively.

#### Depression, Anxiety, Stress Scale-42 (DASS-42)

The Urdu version of Depression, Anxiety and Stress Scale-42 was used. It consisted of 3 subscales; depression, anxiety and stress. In the present study, 2 scales i.e. anxiety and stress were used before and after intervention. Each subscale has 14 items, rated on a 4-point Likert scale. The higher score on each sub-scale, the higher the level of anxiety and stress among participants [18]. The alpha reliability of DASS was reported to be 0.87 [19].

#### Visual Analogue Scale (VAS)

Visual analogue scale was used to analyse the severity of fear for upper GI endoscopy. Participants were asked to rate the intensity of fear on a scale of 0–10. 0 means no fear and 10 means worst fear possible.

### Demographic questionnaire

A semi-structured questionnaire was also designed to obtain basic information regarding participant’s age, gender, education, employment status, marital status, family system, nature of complaints, awareness of diagnosis, type of procedure, history of psychological illness, comorbidity, medicine intake, PPI or H2 blockers, satisfaction status on information provided, do you think endoscope will cause pain when it will go inside, do you think endoscope will damage your internal organs during procedure, do you think endoscope will cause death when it will go inside and fear related to seniority of doctor, etc.

### Statistical analysis

Sample size for the randomized patients was calculated to be over 200 patients (100 in each group), based on significant difference between two groups, keeping type I error (*α*) 0.05, power of 80% and medium effect size of 0.4. Additionally, we estimated that the required number of patients who received upper GI endoscopy was over 250 in consideration of the exclusion criteria.

Analyses were conducted on an intention-to-treat principle where all participants randomized were included. No data substitution was applied to adverse event data. For the primary analysis comparing treatment effects, the least-squares means and their 95% CIs were estimated by one-way analysis of covariance (One-way ANCOVA) with the change in anxiety scores between groups after intervention. This ANCOVA model took into account the variation caused by treatment effects, and baseline DASS score were entered as covariates. Analyses of secondary outcomes were performed in the same manner as the primary analysis. Linear regression analysis (Enter Method) was employed to identify endoscopy-related variables that predict which patients are likely to benefit from visual aids. Quantitative data such as age, income, pre intervention outcome scores were expressed through means and standard deviation. For categorical variables, the Pearson chi-square test and fisher’s exact test were applied. *p* Values <0.05 were considered statistically significant. All analyses were performed using SPSS version 25.0.

## Results

In the present study, there was no significant difference in terms of participant’s age, gender, socio-demographics, endoscopy-related variables and baseline values between conditions as shown in Table 1. Majority of the participants were males (53.4%) in visual aid and (54.3%) no-visual aid group with mean age of 44.01 (SD = 13.29) and 40.37 (SD = 13.20) respectively. Mean income of the participants in video group was Pakistani Rs. 30,025.28 (SD = 16,111.07) and in no-video group was Rs. 24,034.48 (13,884.57). Majority of the participants had education of bachelors, employed, married and belonged to a joint family system in both the groups. 91.4% of the participants’ complaints were chronic in no-video group and 84.5% in video group. Majority of the participants showed an awareness of diagnosis and reported satisfaction over the briefing regarding the endoscopic procedure in both the groups. Majority of the participants were taking medicines, with a significant number of patients on PPI or H2 blockers in both the groups. 24.1% of the participants in no-video group reported that they had comorbid illnesses and 33.6% in video group. Majority of participants of video and no-video group considered that endoscope doesn’t cause pain however, there would be no damage to internal organs or cause death. 77.6% of the participants of no-video group and 57.8% of video group reported no concerns over the seniority of endoscopist (see Table 1).

**Table 1.** Comparison of baseline characteristics among two groups.

	Visual aid Group^a^		No visual aid group^a^		*p* Value
	*f*	%	*f*	%	
Gender					1.00
Female	54	46.6	53	45.7	
Male	62	53.4	63	54.3	
Education					0.481
Illiterate	11	9.5	16	13.8	
Primary	11	9.5	11	9.5	
Middle	7	6.0	13	11.2	
Matric	17	14.7	21	18.1	
Intermediate	5	4.3	3	2.6	
Bachelors	60	51.7	49	42.2	
Masters	5	4.3	3	2.6	
Marital status					0.250
Unmarried	23	19.8	24	20.7	
Married	88	75.9	79	68.1	
Widow	4	3.4	11	9.5	
Divorce	1	0.9	2	1.7	
Employment					0.452
Unemployed	23	19.8	21	18.1	
Employed	71	61.2	65	56	
Self-employed	22	19.0	30	25.9	
Family system					0.235
Nuclear	47	40.5	57	49.1	
Joint	69	59.5	59	50.9	
Nature of complaints					0.157
Acute	18	15.5	10	8.6	
Chronic	98	84.5	106	91.4	
Awareness of diagnosis					0.487
No	42	36.2	36	31	
Yes	74	63.8	80	69	
Type of procedure					1.000
Gastroscopy	88	75.9	88	75.9	
Colonoscopy	28	24.1	28	24.1	
History of psychological illness					1.000
No	112	96.6	112	96.6	
Yes	4	3.4	4	3.4	
Comorbidity					0.147
No	77	66.4	88	75.9	
Yes	39	33.6	28	24.1	
Medicine intake					0.110
No	14	12.1	24	20.7	
Yes	102	87.9	92	79.3	
PPI or H2 blockers					0.499
No	24	20.7	19	16.4	
Yes	92	79.3	97	83.6	
Satisfaction status on information provided					0.748
Satisfied	93	80.2	90	77.6	
Partly satisfied	23	19.8	26	22.4	
Thought about endoscope causes pain when goes inside					0.278
No	39	33.6	48	41.4	
Yes	77	66.4	68	58.6	
Thought about endoscope damages internal organs					0.029
No	65	56.0	82	70.7	
Yes	51	44.0	34	29.3	
Thought about endoscope causes death when goes inside					0.247
No	97	83.6	104	89.7	
Yes	19	16.4	12	10.3	
Fear related to seniority of doctor					0.002
No	67	57.8	90	77.6	
Yes	49	42.2	26	22.4	
	M	SD	M	SD	
Age	44.01	13.29	40.37	13.20	0.130
Income	30,025.28	16,111.07	24,034.48	13,884.57	0.003
Pre-intervention anxiety	11.41	2.26	11.59	2.24	0.541
Pre-intervention stress	21.29	2.50	21.06	2.65	0.508
Pre-intervention fear	7.35	1.53	7.86	1.49	0.010

^a^*N* = 232 (*n* = 116 for each group). For categorical variable, *p*-value was based on Pearson chi-square test and fisher’s exact test, and for continuous variable, *p* value is based on *t*-test.

One-way ANCOVA was conducted to determine statistically significant difference between interventions on post-intervention anxiety scores after controlling for pre-intervention anxiety scores. The covariate, pre-intervention anxiety, was significantly related to post-intervention anxiety, *F*(1, 229) = 1088.36, *p* < .001, partial *η*^2^ = .83. There was also a significant effect of intervention on post-anxiety scores after controlling for the effect of pre-anxiety scores, *F*(1, 229) = 1605.21, *p*< .001, partial *η*^2^ = 0.88. The adjusted and unadjusted means are presented in Table 2. Planned contrasts revealed that counselling with visual aid of endoscopy procedure (*M* = 4.51, SE = .08) significantly decreased anxiety compared to counselling with no-visual aid (*M* = 8.77, SE = 0.08) condition, *t*(229) = 40.07 *p* < .001, 95% CI (−4.46, −4.05).

**Table 2.** Unadjusted and covariate adjusted descriptive statistics of primary and secondary outcomes for interventions by analysis of covariance.

	Before intervention			After intervention (unadjusted)		After intervention (adjusted)	
Interventions	*N*	Mean	SE mean	Mean	SE mean	Mean	SE mean
Anxiety (primary outcome)							
Video	232	11.41	0.21	4.44	0.16	4.51	0.08
No-video	232	11.59	0.21	8.84	0.19	8.77	0.08
Stress (secondary outcome)							
Video	232	21.29	0.23	11.80	0.23	11.69	0.10
No-video	232	21.06	0.25	16.94	0.26	17.05	0.10
Fear (secondary outcome)							
Video	232	7.35	0.14	2.46	0.10	2.65	0.05
No-video	232	7.86	0.14	5.66	0.13	5.47	0.05

The covariate, pre-intervention stress score, was significant, *F*(1, 229) = 1168.42, *p* < .001, partial *η*^2^ = .84, indicating that stress scores before intervention had a significant effect on stress scores after intervention. There was also a significant effect of visual aid on post-intervention stress scores after controlling for the effect of pre-intervention stress scores, *F*(1, 229) = 1424.49, *p*< .001, partial *η*^2^ = 0.86. The adjusted and unadjusted means are presented in Table 2. Planned contrasts revealed that video of endoscopy procedure along with counselling prior to endoscopy (*M* = 11.69, SE = .10) significantly decreased stress compared to counselling with no-visual aid (*M* = 17.05, SE = 0.10) condition, *t*(229) = 37.74 *p* < .001, 95% CI (−5.63, −5.07).

The covariate, pre-intervention fear score, was significant, *F*(1, 229) = 904.99, *p* < .001, partial *η*^2^ = .79, indicating that fear scores before intervention had a significant effect on fear scores after intervention. There was also a significant effect of visual aid on post-fear scores after controlling for the effect of pre-fear scores, *F*(1, 229) = 1438.13, *p* < .001, partial *η*^2^ = 0.86. The adjusted and unadjusted means were presented in Table 2. Planned contrasts revealed that visual aid along with counselling (*M* = 2.65, SE = .05) significantly decreased fear of endoscopy compared to counselling with no-visual aid (*M* = 5.47, SE = 0.05) condition, *t*(229) = 37.92 *p* < .001, 95% CI (−2.97, −2.67).

Results of linear regression analyses (Enter Method) showed that gender was significant negative predictor of stress which means that male patients were less stressed after visual aid intervention as compared to female patients. Patients who had chronic GI symptoms were less stressed after visual aid intervention as compared to those who had acute GI symptoms. Satisfaction on briefing of endoscopy procedure was significant positive predictor of stress and fear which means that patients who were fully satisfied of briefing were less stressed and feared after visual aid intervention as compared to those who were partly satisfied. Patients who had concern over seniority of endoscopist who was going to conduct the procedure were less feared of endoscopy procedure after visual aid intervention as compared to those who had no concerns. However, age, history of psychological illness, thoughts about endoscope causes pain and damage to internal organs did not emerge as significant predictors (see Table 3).

**Table 3.** Multiple regression for endoscopy-related variables as predictors of post-intervention anxiety, stress and fear.

Variables	Anxiety				Stress				Fear			
	*B*	*SE B*	*β*	95% CI	*B*	*SE B*	*β*	95% CI	*B*	*SE B*	*β*	95% CI
Constant	3.75	1.05		1.67, 5.84	13.19	1.44		10.34, 16.04	2.61	0.64		1.34, 3.87
Age	0.02	0.01	.14	−.01, .04	0.02	0.02	.13	−.01, .05	0.01	0.01	.13	−.01, .02
Gender^a^	−0.33	0.33	−.09	−.99, .33	−0.87	0.45	−.17*	−1.77, .03	−0.31	0.20	−.14	−.71, .09
Nature of complaints^b^	−0.13	0.46	−.03	−1.05, .74	−1.58	0.63	−.23*	−2.84, −.33	−0.44	0.28	−.15	−1.00, .12
History of psychological illness^c^	−1.66	0.92	−.17	−3.48, .15	−1.25	1.25	−.09	−3.74, 1.23	−0.78	0.56	−.13	−1.88, .32
Comorbidity^c^	−0.14	0.36	−.04	−.86, .58	−0.58	0.49	−.11	−1.56, .41	−0.12	0.22	−.05	−.56, .32
Satisfaction on briefing^d^	0.72	0.43	.16	−.13, 1.56	1.64	0.58	.26**	.48, 2.79	0.61	0.26	.23*	.09, 1.12
Endoscope causes pain^c^	−0.06	0.43	−.02	−.90, .78	−0.39	0.58	−.07	−1.54, .76	−0.08	0.26	−.03	−.59, .43
Endoscope damages internal organs^c^	−0.03	0.45	−.01	−.92, .87	0.22	0.62	.04	−1.00, 1.44	−0.04	0.27	−.02	−.58, .51
Endoscope causes death^c^	−0.18	0.47	−.04	−1.13, .76	−1.01	0.65	−.15	−2.29, .28	0.06	0.29	.02	−.52, .63
Seniority of doctor^c^	−0.67	0.36	−.19	−1.37, .03	−0.79	0.48	−.16	−1.75, .17	−0.44	0.22	−.20*	−.87, −.01
*R* ^2^	0.11				0.20				0.14			
*F*	1.28				2.54**				1.72			

*Note. N =* 116. *p< .05. **p < .01. ^a^Female = 0, male = 1. ^b^acute = 1, chronic = 2. ^c^No = 0, Yes = 1. ^d^Satisfied = 1, partly satisfied = 2.

## Discussion

The study investigated the impact of an endoscopic procedure on anxiety, fear and acute stress levels in patients undergoing the procedure. The participants were provided detailed information about the relevant procedure coupled with psychological counselling with or without visual aid in the form of a short-animated video of the endoscopy procedure (a psycho-intervention).

In the present study, results revealed that there was a significant post-intervention scores difference in visual aid and no visual aid condition with respect to anxiety, stress and fear i.e. video group has significantly reduced anxiety, stress and fear as compared to no-video group. This can be related to a study done by Murugesan et al. who reported a statistically significant difference between video or verbal information group after endoscopy i.e. information provided by video helps reduce anxiety of the patients [11]. Similarly, Kannan et al. reported significance difference in experimental (music) and control group (no music) i.e. patients who listened to music had decreased post-anxiety levels as compared to those who didn’t listen [12]. Similar findings were reported in patients undergoing uterine endoscopy (hysteroscopy) and stressed the importance of establishing pharmacological and non-pharmacological tools to alleviate stress and anxiety in patients undergoing endoscopic procedures [20,21]. These findings are new in the present study as most of the available literature is related to anxiety among patients undergoing upper GI endoscopy but none of them addressed efficacy of showing animated video of endoscopy prior to procedure on reducing anxiety, fear and stress, which needs to be further explored.

Present study findings revealed that gender, chronic GI symptoms, concern over seniority of endoscopist were emerged as significant predictors of stress. This can be related to the findings of a study which showed that gender was a significant predictor of anxiety prior to endoscopy [10] i.e. females reported more concern about endoscopy procedure as compared to men [22]. There was association between stress and GI diseases [23]. During this study, experience and seniority level of endoscopist was considered and it was established that patients’ stress and anxiety scores had a relation with the seniority of the endoscopist as evidenced in other studies [24].

It was also found in the present study that patients who were fully satisfied of briefing of endoscopy procedure were less stressed and feared after visual aid intervention as compared to those who were partly satisfied. Umezawa et al. reported that patients who received relaxing visual distraction reported significantly higher post-procedure satisfactory levels as compared to patients who didn’t receive relaxation method [15]. Sogabe et al. also reported in a study that satisfaction from distraction in audio-visual group was high as compared to audio distraction or visual distraction alone [14].

In the present study, results also revealed that age, history of psychological illness, endoscope causes pain and damage to internal organs did not emerge as significant predictors. This can be supported by a study done by Volkan et al. who reported no significant difference between group whom procedure was explained in detailed and those who were briefly explained in terms of age [25]. In another study, it was found that there was no significant relation of age and history of psychiatric illness with anxiety level [26]. The relationship of pain or possible damage during a medical procedure has been extensively studied and the data suggested that this can lead to an increase in anxiety sensitivity in the pre- or post-procedural settings [27]. However, this was not a statistically significant predictor for visual aid group.

## Conclusion

Endoscopic procedures are associated with an increase in anxiety and acute stress levels. This increase in anxiety, fear and acute stress can be alleviated with psychological counselling coupled with a video animation before the procedure however, visual aid does confer superiority over counselling; the introduction of visual aid resulted in additional benefits by relieving the stress and fear or an improvement in the anxiety levels of the patients. Moreover, a number of factors (such as gender, nature of complaints, concern over seniority of endoscopist and satisfaction on briefing of endoscopy procedure) were identified that predict which patients were likely to benefit from visual aids, and thus help selection.

## Limitations

The study did not consider the type of procedure an individual patient was undergoing, the difference in steps and length of gut preparation methods and the estimated time of the particular procedure.

This was a single centre, experimental study conducted in patients who were undergoing similar procedures (gastroscopy or colonoscopy) albeit with the addition of interventions in some patients that can have an impact on the anxiety, fear or acute stress scores. Although, the patients were shown a brief animation of the respective procedure after a counselling session but they were not requested to perform a relaxation exercise to evaluate and compare the effect of relaxation techniques versus counselling alone in patients undergoing medical or surgical procedures. A multicentre, experimental study can be planned to study whether a 5-min relaxation exercise can help alleviate the anxiety, acute stress or fear further or counselling along with video of procedure is beneficial for the patients undergoing any medical or surgical intervention.

## Clinical implications

This study can help in improving the pre-procedure guidelines to reduce the anxiety, fear and stress levels among patients undergoing diagnostic or therapeutic endoscopic procedures along with the standard of healthcare. It will help to improve standard operating procedures before an invasive procedure and afford more comfort to the patient. It is a safe, low-cost nonpharmacological method that may play an important role in improving physical and psychological factors before the endoscopic procedure. The endoscopists should implement counselling with visual aid intervention to reduce the anxiety and distress towards endoscopy and enhance patient cooperation during the procedure.

Future studies can be done after the implementation of the updated standard operating procedures comparing the impact of pharmacological and non-pharmacological interventions to reduce anxiety and stress in patients undergoing endoscopy. A randomized control trial can be conducted to compare the efficacy of pharmacological agents versus psychoeducation and the use of drugs prior to the endoscopy procedure can be reduced.

## Data Availability

The data that support the findings of this study are available on request from the corresponding author.
